# Parent Emotion Socialization Behaviors and Adolescent Psychological Symptoms in Families Impacted by Tourette Syndrome

**DOI:** 10.1007/s10802-025-01393-z

**Published:** 2026-02-03

**Authors:** Abigail L.B. Snow, Isabelle Taylor, Brandon Low, David A. Isaacs, Daniel O. Claassen, Kelly H. Watson

**Affiliations:** 1 Department of Psychology & Human Development, Vanderbilt University, Nashville, TN USA; 2 Department of Neurology, Vanderbilt University Medical Center, Nashville, TN USA

**Keywords:** Tourette syndrome, Neurodevelopmental disorders, Emotion socialization, Parenting, Adolescent psychopathology

## Abstract

Parent emotion socialization (ES) behaviors, encompassing supportive and unsupportive responses to adolescent emotions, are an important factor in shaping youths’ psychological development, but their impact in adolescents with Tourette syndrome (TS) has been largely overlooked. While tics are the defining symptom of TS, psychological comorbidities are widespread in this population and more strongly predict quality of life than tic severity. As such, there is an urgent clinical need to identify risk factors for psychopathology in adolescents with TS. Parent ES behaviors in families affected by TS have not yet been examined, but evidence of (a) the impact of parent ES behaviors in other neurodevelopmental disorders and (b) environmental sensitivity in TS suggests adolescents with TS may be particularly sensitive to parent ES behaviors. The current study utilized behavioral observations of parent-adolescent dyads (*n* = 29) engaging in a conflict discussion to examine ES behaviors in parents of adolescents with TS. Additionally, we investigated the relationship of observed ES behaviors with adolescent self-reported psychological symptoms. Results revealed that parents of adolescents with TS engaged in wide ranges of both supportive and unsupportive ES behaviors. Further, greater use of supportive ES behaviors was significantly related to fewer internalizing and externalizing symptoms in adolescents, and greater use of unsupportive ES behaviors was significantly related to greater internalizing, and marginally related to greater externalizing, symptoms in adolescents. Findings highlight the importance of parental support in this population and implicate parent ES as a novel target for intervention in families affected by TS.

Adolescence marks a critically important developmental period characterized by transitions to increased independence and autonomy, mastery of self-regulation skills, and, for many individuals at risk, the emergence of psychopathological symptoms (Dalsgaard et al., 2020). Despite individuals’ growing independence during this period, parents maintain an important role in shaping youths’ utilization of regulatory strategies and influencing their subsequent psychosocial trajectories (Morris et al., 2007, 2017). Parent emotion socialization (ES) behaviors in particular have been related to psychological outcomes in children (Eisenberg, 2020); however, their impact on adolescent outcomes has been largely unexamined. Additionally, while some work has focused on youth with neurodevelopmental conditions (e.g., Breaux et al., 2018; Jordan et al., 2021), much of the work on parent ES behaviors has focused on typically developing children (see Eisenberg, 2020; Eisenberg et al., 1998 for reviews). Tourette syndrome (TS) is a prime example of a neurodevelopmental disorder that places adolescents at heightened risk for psychosocial difficulties, and there is a critical need to understand the influence of parent ES behaviors in adolescents with TS to identify potential novel targets for intervention in this at-risk population. Parent ES describes the method through which parents educate their children about the experience, expression, and regulation of emotions and emotion-related behavior (Eisenberg et al., 1998). Such parenting behaviors involve reactions to children’s emotions, discussions of emotions, and expressions of emotions, which entail both direct and indirect mechanisms of influencing children’s emotional understanding. ES behaviors have been conceptually grouped into two broad categories: supportive and unsupportive (Eisenberg, 2020; Eisenberg et al., 1998). Supportive ES behaviors include validation, comfort, and education about emotions and teaching strategies to regulate them. Supportive ES behaviors have been shown to be related to better emotion regulation (Cui et al., 2020; Perry et al., 2020) and fewer internalizing and externalizing problems in youth (Johnson et al., 2017; McKee et al., 2022; Thompson et al., 2020). In contrast, unsupportive ES behaviors include punitive, minimizing, dismissive, rejecting, or personal distress reactions towards emotions. Unsupportive ES behaviors have been shown to be related to poor neurophysiological markers of regulation (Garcia et al., 2024; McKee et al., 2022; Perry et al., 2020; Tan et al., 2020) and greater internalizing and externalizing problems in youth (Cui et al., 2020; Johnson et al., 2017; McKee et al., 2022). Multiple intervention studies have shown that increasing supportive and decreasing unsupportive ES behaviors leads to better emotional competence and regulation, improved behavioral adjustment, and reduced internalizing and externalizing symptoms in children (Eisenberg, 2020; England-Mason et al., 2023; Kehoe et al., 2014; Watson et al., 2014). Despite the well-known contributions of parenting to youth outcomes in neurotypical populations, parent ES behaviors in TS have not yet been examined.

TS is a neurodevelopmental disorder characterized by sudden, brief, repetitive, non-rhythmic movements (i.e., motor tics) and vocalizations (i.e., vocal tics) with childhood onset and persistence for at least one year (American Psychiatric Association, 2013), impacting about 1% of the population (Knight et al., 2012; Robertson, 2008). TS is more common in males than females, diagnosed at a ratio of about 4:1 (Ludolph et al., 2012). Tics typically begin around the ages of 4 to 6, peak in severity during early-to-middle adolescence, and decrease in severity in late adolescence, with rare remission in adulthood (Black et al., 2021; Bloch & Leckman, 2009; Groth et al., 2017; Reagan et al., 2022). Adolescents with TS are at heightened risk for psychosocial difficulties, including psychological problems, poorer social functioning, and decreased quality of life (Cutler et al., 2009; Eapen et al., 2016; Eddy, Cavanna, et al., 2011; Lee et al., 2024). While tics are the defining symptom of TS, the vast majority of individuals with TS experience psychological comorbidities across both internalizing and externalizing spectrums (Hirschtritt et al., 2015). Symptoms of depression, anxiety, obsessive-compulsive disorder (OCD), attention-deficit/hyperactivity disorder (ADHD), defiance behaviors, conduct problems, and rage attacks are present at higher rates amongst youth with TS (Mol Debes, 2013; Robertson, 2006). More severe emotion dysregulation and comorbid psychopathology are related to greater tic severity and poorer quality of life in individuals with TS across the lifespan (Eapen et al., 2016; Eddy, Cavanna, et al., 2011; Huisman-van Dijk et al., 2019; Ramsey et al., 2020). Current behavioral and pharmacological treatments in TS populations tend to focus on tic management, even though psychological symptoms are often the most impairing symptoms (Dooley et al., 1999; Ghanizadeh et al., 2010) and have consistently been shown to be a stronger predictor of quality of life than tic severity (e.g., Eddy, Rizzo, et al., 2011; Watson et al., 2024). As such, there is an urgent clinical need to identify modifiable risk factors for psychopathology in TS. Parent ES behaviors may provide an additional intervention target to improve clinical outcomes in adolescents with TS.

While no studies to date have assessed parent ES behaviors in families impacted by TS, there are several reasons to hypothesize that the way parents socialize emotions may have particular relevance in this clinical population. First, emotion dysregulation, poor inhibitory control, and psychopathology, each of which are more prevalent in TS (Hirschtritt et al., 2015; Hovik et al., 2015; Morand-Beaulieu et al., 2017), have been associated with parent ES behaviors in other populations (Root & Rasmussen, 2017; Thompson et al., 2020). Second, higher levels of parental stress and psychopathology and more negative parental perceptions of their children, all of which are evident in parents of youth with TS (Cooper et al., 2003; Goussé et al., 2016; Lee et al., 2007; Leivonen et al., 2017; Robinson et al., 2013), have been related to fewer supportive and greater unsupportive parent ES behaviors in other populations (e.g., Breaux et al., 2016; Edler & Valentino, 2024; Root & Rasmussen, 2017). Lastly, based on evidence from studies in other neurodevelopmental populations, children with specific psychological symptoms (e.g., anxiety, ADHD symptoms) may be more sensitive to the impact of parent ES behaviors than their neurotypical peers (Breaux et al., 2018; Hurrell et al., 2017; Jordan et al., 2021). Although sensitivity to parenting behaviors has not specifically been examined in TS, individuals with TS may be more sensitive to their environment generally, based on evidence of sensory hypersensitivity (Isaacs et al., 2022; Isaacs & Riordan, 2020) and the impact of stress and contextual factors on tic severity (Conelea & Woods, 2008; Lin et al., 2007). As such, adolescents with TS may be particularly receptive to supportive ES behaviors and/or vulnerable to unsupportive ES behaviors.

The current study sought to characterize parent ES behaviors in the context of families impacted by TS and investigate the relationship between observed parent ES behaviors and adolescent self-reported psychological symptoms. First, we hypothesized that parents of adolescents with TS would be observed to use both supportive and unsupportive ES behaviors in response to their adolescent’s negative emotions. Second, we hypothesized that greater use of supportive ES behaviors would be related to fewer adolescent self-reported internalizing and externalizing symptoms and that greater use of unsupportive ES behaviors would be related to greater adolescent self-reported internalizing and externalizing symptoms.

## Method

### Participants

Participants included 32 adolescents (ages 11–17) who met *Diagnostic and Statistical Manual of Mental Disorders, 5th edition* (DSM-5; American Psychiatric Association, 2013) criteria for Tourette’s Disorder or another chronic tic disorder (collectively referred to as ‘adolescents with TS’). Three participants were missing behavioral observation data due to time constraints (*n* = 1) or technical error (*n* = 2). Therefore, the final dataset included 29 adolescents with TS (*M* age = 13.93, *SD* = 2.20; 31% female). Adolescents were predominantly White (79%) and non-Hispanic (83%). A majority (62%) of adolescents were prescribed medication, with most medications serving as treatment for tics, ADHD, and/or anxiety. Adolescents had a range of comorbid psychiatric diagnoses, including anxiety (38%), ADHD (28%), OCD (24%), and depression (7%). All adolescents participated with an adult caregiver who was their legal guardian (collectively referred to as ‘parents’). Parents included 23 mothers, 5 fathers, and 1 grandmother (83% female). Parents were predominantly White (93%) and non-Hispanic (93%).

### Procedure

Adolescents with TS were recruited from a Tourette Association of America Center of Excellence clinic in the southeastern United States. Recruitment and enrollment occurred from September 2023 to March 2025. Inclusion criteria included a child 11–17 years old, a parent or legal guardian willing to complete caregiver assessments, English proficiency, and a diagnosis of TS or chronic tic disorder per DSM-5 criteria in the adolescent. Exclusion criteria included uncorrected vision impairment or a diagnosis of autism spectrum disorder (ASD), intellectual disability, or schizophrenia in the adolescent. All adolescents recruited were being seen for follow-up at the clinic, with at least one prior visit to establish care for tics.

The study procedures occurred during a single visit and were part of a larger study examining social and cognitive processes in TS. Adolescents completed paper-based questionnaires while separated from their parent. Then, dyads were brought together to complete a semi-structred interview about tics and a behavioral observation task. Parents and their adolescents were instructed to discuss a source of conflict or disagreement for 10 minutes while being video-recorded. The dyad was given a task card to help guide the discussion, which instructed them to: 1) explain how they each feel about the issue, 2) discuss why it is a source of conflict or disagreement, and 3) try to reach a solution. The study was reviewed and approved by the Institutional Review Board at Vanderbilt University Medical Center (#231090). Parents provided written informed consent and adolescents provided written informed assent in-person prior to study participation. Parents consented for their own and their adolescent’s participation and adolescents assented to participate. Participants were compensated after completion of the study visit.

### Measures

#### Tic Severity

The Yale Global Tic Severity Scale (YGTSS; Leckman et al., 1989) is a semi-structured interview that was administered by trained members of the research team to parents and adolescents to assess adolescents’ tic severity and impairment in the past week. The YGTSS is the gold-standard rating scale to quantify tic severity. The Total Tic Severity score ranges from 0 to 50, with higher scores indicating greater severity. Scoring interpretation guidelines (McGuire et al., 2021) suggest the following cut-offs to classify severity: 1–6 (*Borderline*), 7–10 (*Mild*), 11–27 (*Moderate*), 28–43 (*Marked*), 43–46 (*Severe*), 47–50 (*Extreme*).

#### Emotion Socialization

Behavioral codes for parent ES behaviors were developed to address limitations of existing coding systems, namely that current schemes are adapted upward from schemes developed for young children or disagree on how to capture broad factors of supportive and unsupportive ES behaviors. Parent ES behaviors were measured via coding of behavioral observations of the parent-adolescent conflict discussion task. Parent ES codes were developed guided by the ES literature and the Iowa Family Interaction Rating Scales (IFIRS; Melby & Conger, 2000). IFIRS is a macro-level behavioral coding system designed to assess individual and dyadic emotional and behavioral aspects of interaction, including verbal and non-verbal communication. Codes are assigned values from 1 (*not at all characteristic*) to 9 (*mainly characteristic*) based on the frequency and intensity of behavior. Parents were rated on two codes based on ES behaviors in response to their adolescent’s expressed negative emotions: supportive ES behaviors (SESB) and unsupportive ES behaviors (UESB). Table 1 includes definitions and examples for each code. SESB included behaviors that were validating, accepting, supportive, open to discussion, encouraging, and child-centered. This included parents’ elaboration, affirmation, solicitation, explanation, and coaching in response to adolescents’ negative emotions. UESB included behaviors that were dismissive, minimizing, punitive, blaming, or rejecting. This included parents’ invalidation, minimization, punishment, criticism, or harsh discipline in response to adolescents’ negative emotions. Each video was viewed three times and independently rated by two trained coders who met to establish consensus on any discrepant codes differing by more than two points. Codes that differed by only one point were given the higher score and were considered reliably coded (Melby & Conger, 2000). Coders established consensus on 97% of the videos. The percentage of agreement between coders prior to establishing consensus was calculated to determine interrater reliability and was acceptable (71% for SESB and 75% for UESB).

**Table 1:** Definitions and examples of emotion socialization codes

Emotion Socialization Code	Definition	Examples
Supportive Emotion Socialization Behaviors (SESB)	The extent to which the parent’s interactions in response to the child’s display of negative emotion are validating, accepting, supportive, sensitive, open to discussion, encouraging, and child-centered. The parent displays an awareness of the child’s emotion and responds with an attitude that views emotional display as an opportunity for bonding or teaching and encourages emotional expression and reflection.	1. “I’m sorry. That sounds really stressful.” 2. “What could we do to help calm down? What if we try to take some space?” 3. “I know how hard it was to go through that. Would you say you came out of it stronger?” 4. “What parts of it do you think made you feel sad? Why did they do that?” 5. Parent gives crying child a hug.
Unsupportive Emotion Socialization Behaviors (UESB)	The extent to which the parent’s interactions in response to the child’s display of negative emotion are dismissive, minimizing, invalidating, insensitive, punitive, blaming, or rejecting. The parent may attempt to minimize the child’s emotion or close off emotional discussion by responding in a manner that is self-focused, critical, or harsh and conveys a belief that negative emotions are harmful, must be fixed or changed quickly, and are fleeting or unimportant.	1. “Stop crying or go to your room!” 2. “It’s not that big of a deal. You’re being overdramatic about it.” 3. “Don’t freak out too much. It makes me anxious.” 4. “I can’t stand it when you’re mad.” 5. “We wouldn’t be in this situation if you didn’t get so angry.”

#### Internalizing and Externalizing Symptoms

Adolescent symptoms of psychopathology were assessed using the Internalizing Problems and Externalizing Problems subscales of the Youth Self Report (YSR; Achenbach & Rescorla, 2001). The YSR includes a 112-item checklist of psychological symptoms and behaviors. Adolescents were asked to rate how much each statement described themselves in the past 6 months on a 3-point scale (0 = *not true*; 1 = *sometimes or somewhat true*; 2 = *very true or often*). The Internalizing Problems (31 items) and Externalizing Problems (32 items) subscales produce normative *T* scores (*M* = 50, *SD* = 10) derived from a nationally representative sample of adolescents. Internal consistencies for the Internalizing (*α* = 0.91) and Externalizing (*α* = 0.82) Problems subscales in the current sample were good.

### Statistical Analyses

All statistical analyses were computed using SPSS (version 30). Descriptive statistics were calculated for all measures (see Table 2). Associations between age, tic severity, parent ES behaviors, and adolescent psychological symptoms were examined using bivariate Pearson correlations. Independent samples *t*-tests were used to determine sex differences in all key study variables. A paired samples *t*-test was used to examine within-parent differences in use of ES behaviors. Linear regression analyses were conducted to examine the relationship between parent ES behaviors and adolescent internalizing and externalizing symptoms. Raw scores on the Internalizing and Externalizing Problems subscales were used as dependent variables, although the *T* scores are presented in Table 2 to allow for meaningful comparisons to normative data. Adolescent sex was included as a covariate in all analyses. All variables met regression assumptions. Residuals followed an approximately normal distribution and were independent of each other. In reflection of the directionality of hypotheses, all analyses used one-tailed tests (*p* < 0.05) to determine statistical significance.

**Table 2:** Descriptive statistics for all study variables

Variable	*M* (*SD*)	Range
Adolescent Age (years)	13.93 (2.20)	11–17
Female Sex [*n* (%)]	9 (31%)	–
YGTSS Total Tic Severity	21.62 (8.62)	5–42
Supportive Emotion Socialization (SESB)	5.69 (1.63)	3–9
Unsupportive Emotion Socialization (UESB)	4.14 (1.90)	1–9
YSR Internalizing Problems		
Raw score	16.00 (11.15)	0–45
*T* score	57.34 (13.38)	30–83
YSR Externalizing Problems		
Raw score	9.55 (6.05)	1–25
*T* score	50.03 (8.58)	34–68

*SESB* Supportive Emotion Socialization Behaviors; *UESB* Unsupportive Emotion Socialization Behaviors; *YGTSS* Yale Global Tic Severity Scale; *YSR* Youth Self Report

## Results

### Descriptive Statistics

Descriptive statistics are shown in Table 2. Adolescents had a wide range of Total Tic Severity scores, with a mean total score in the moderate severity range (*M* = 21.62, *SD* = 8.62). Further, 10% of adolescents fell in the borderline-to-mild range (scores 1–10), 66% fell in the moderate range (scores 11–27), and 24% fell in the marked range of tic severity (scores 28–43). No adolescent had severe or extreme tic severity. Additionally, the sample exhibited a wide range of internalizing and externalizing symptoms. Notably, while the sample mean was not elevated on YSR Externalizing Problems (*M* = 50.03, *SD* = 8.58), the sample mean for YSR Internalizing Problems was over half a standard deviation above the normative mean (*M* = 57.34, *SD* = 13.38). Additionally, there was a significant difference between sexes on YSR Internalizing Problems both on raw score (*t*(13.92) = 2.94, *p* = 0.005) and *T* score (*t*(27) = 1.84, *p* = 0.038), such that girls self-reported significantly greater internalizing symptoms than boys. There were no other significant differences between sexes.

### Characteristics of Parent Emotion Socialization Behaviors

**Fig. 1:**
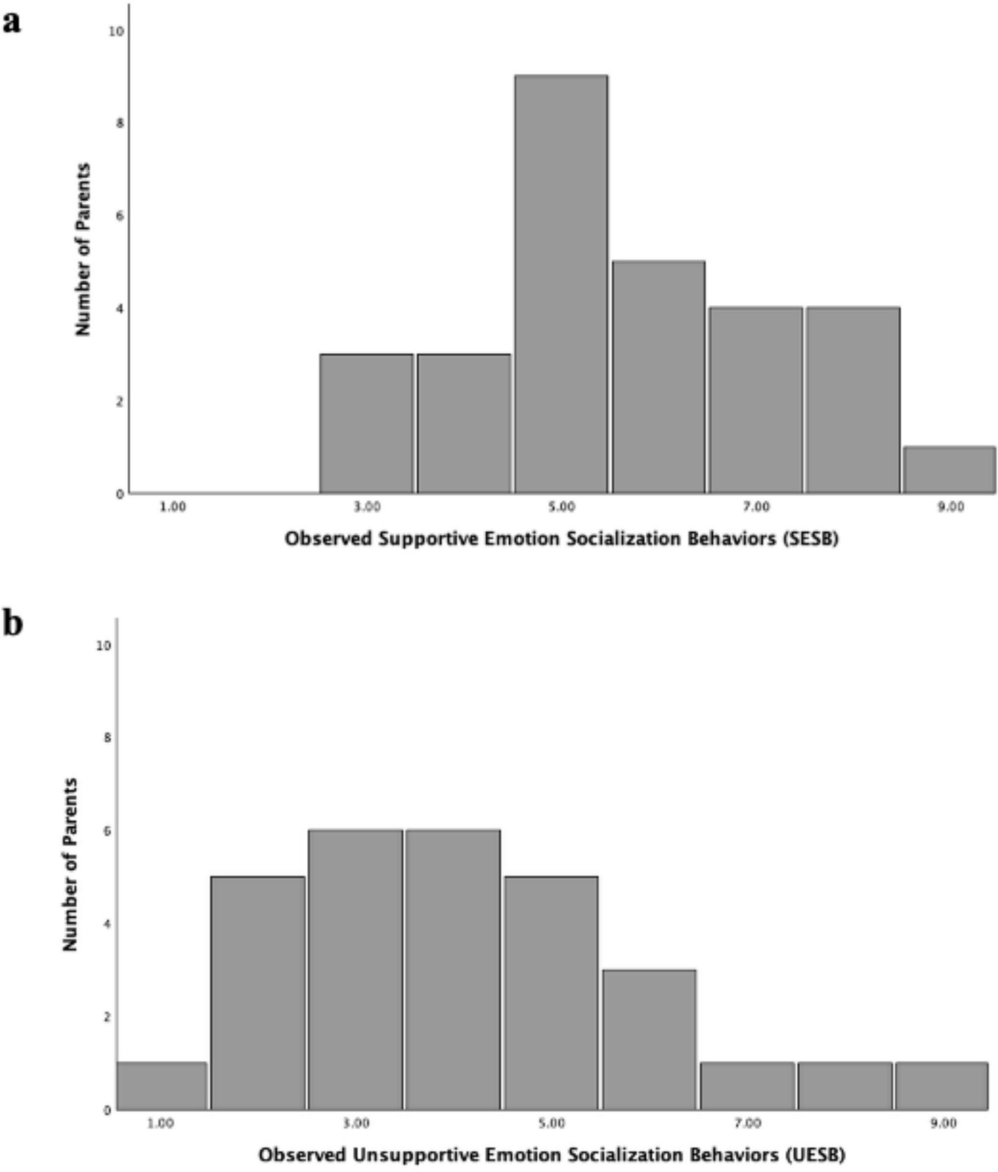
Frequency of parent observed **a**) supportive emotion socialization behaviors (SESB) and **b**) unsupportive emotion socialization behaviors (UESB) codes

Histograms of observed ES behavior codes are shown in Fig. 1. Parents were observed to engage in a wide range of both SESB and UESB. All parents were observed to use at least minimal evidence of SESB, with a minimum of 3, and SESB tended to cluster toward the high-center of the range. Observations of parents’ UESB spread across the full range from 1 to 9, though were clustered toward the low-center of the range. Parents’ mean observed use of ES behaviors are shown in Table 2. The mean for SESB (*M* = 5.69, *SD* = 1.63) corresponded to a code of *Somewhat Characteristic*, while the mean for UESB (*M* = 4.14, *SD* = 1.90) fell between codes of *Minimally* and *Somewhat Characteristic*. Parents engaged in significantly more SESB than UESB (*t*(28) = 2.97, *p* = 0.003). There were no differences in ES behaviors based on adolescent sex.

### Relationships between Parent Emotion Socialization Behaviors and Adolescent Psychological Symptoms

**Fig. 2:**
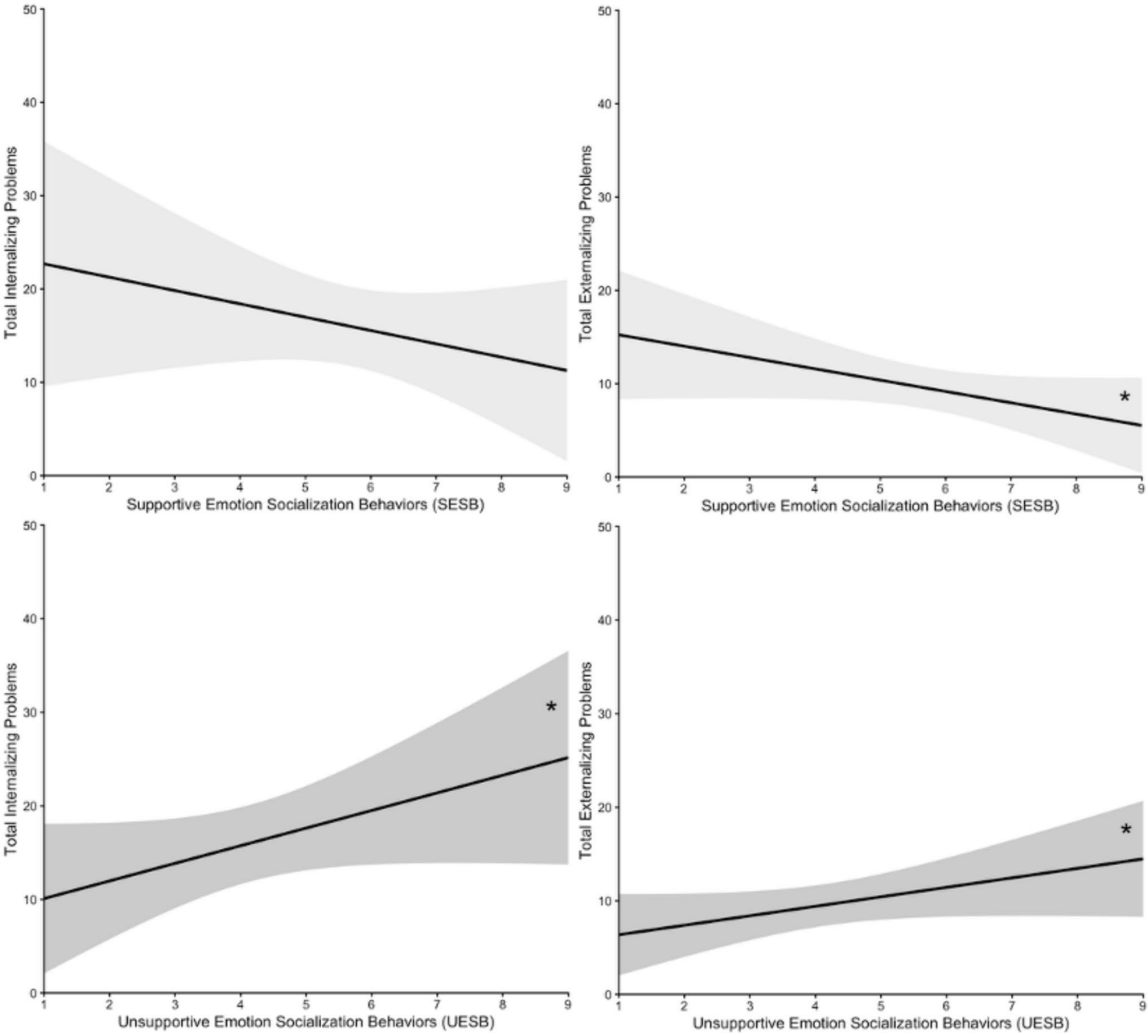
Relationships between observed supportive emotion socialization behaviors (SESB; light gray; top) and unsupportive emotion socialization behaviors (UESB; dark gray; bottom) with adolescent self-reported total internalizing (left) and externalizing (right) symptoms. *p < 0.05

Bivariate correlations are presented in Table 3. Adolescent age was significantly related to UESB (*r* = 0.34, *p* = 0.034), such that parents tended to use more UESB when their adolescents were older. Tic severity was not related to any variables. SESB and UESB were marginally related (*r* = −0.26, *p* = 0.085), such that greater use of SESB was related to lower use of UESB. Greater use of SESB was significantly associated with fewer externalizing symptoms (*r* = −0.33, *p* = 0.042), and greater use of UESB was significantly associated with greater internalizing (*r* = 0.32, *p* = 0.045) and externalizing (*r* = 0.32, *p* = 0.046) symptoms. The effect sizes for the significant bivariate correlations were small to medium in magnitude (Cohen, 1988). Figure 2 summarizes the relationships between ES behaviors and adolescent psychological symptoms.

**Table 3:** Bivariate correlations amongst key study variables

Variable	1	2	3	4	5	6
1. Adolescent Age	–					
2. YGTSS Total Tic Severity	0.18	–				
3. Supportive Emotion Socialization (SESB)	−0.08	0.12	–			
4. Unsupportive Emotion Socialization (UESB)	0.34*	−0.07	−0.26^+^	–		
5. YSR Internalizing Problems	0.11	0.05	−0.21	0.32*	–	
6. YSR Externalizing Problems	0.12	0.09	−0.33*	0.32*	0.69**	–

*SESB* Supportive Emotion Socialization Behaviors; *UESB* Unsupportive Emotion Socialization Behaviors; *YGTSS* Yale Global Tic Severity Scale; *YSR* Youth Self Report

**p < 0.001, *p < 0.05, ^+^*p* < 0.10

Linear regression analyses are presented in Table 4. Adolescent sex was included as a covariate in all four models due to the higher prevalence of males in our sample and the sex difference in internalizing symptoms. Adolescent sex was a significant predictor of internalizing symptoms in both models (*p*’s < 0.01), and marginally predicted externalizing symptoms in Model 1 (*p* = 0.056), such that females were more likely to self-report higher levels of symptoms. After controlling for adolescent sex, greater use of SESB significantly predicted fewer internalizing (*β* = −0.34, *p* = 0.043) and externalizing symptoms (*β* = −0.41, *p* = 0.030), and greater use of UESB significantly predicted greater internalizing symptoms (*β* = 0.33, *p* = 0.047) and marginally predicted greater externalizing symptoms (*β* = 0.32, *p* = 0.083). The effect sizes were medium in magnitude (Cohen, 1988).

**Table 4:** Linear regression analyses predicting adolescent psychological symptoms from parent emotion socialization behaviors

Model	Dependent Variable			
	YSR Internalizing Problems		YSR Externalizing Problems	
	*β*	*p*	*β*	*p*
Model 1	(*F* = 7.62**, *R*^*2*^ = 0.37)		(*F* = 3.79*, *R*^*2*^ = 0.23)	
Adolescent Sex	−0.59**	0.00	−0.35^+^	0.06
Observed SESB	−0.34*	0.04	−0.41*	0.03
Model 2	(*F* = 7.52**, *R*^*2*^ = 0.37)		(*F* = 2.72^+^, *R*^*2*^ = 0.17)	
Adolescent Sex	−0.51**	0.00	−0.27	0.15
Observed UESB	0.33*	0.05	0.32^+^	0.08

*SESB* Supportive Emotion Socialization Behaviors; *UESB* Unsupportive Emotion Socialization Behaviors; *YSR* Youth Self Report. For adolescent sex, female = 1 and male = 2

**p < 0.01; *p < 0.05; ^+^*p* < 0.10

## Discussion

The current study characterized parent ES behaviors in families of adolescents with TS and examined the assocations between observed parent ES behaviors with adolescent self-reported psychological symptoms. To our knowledge, this is the first study to examine parent ES behaviors in TS, extending the scant amount of research on ES behaviors in other neurodevelopmental populations (e.g., ADHD, ASD; Breaux et al., 2018; Jordan et al., 2021) to TS. Additionally, the current study adds to the limited body of ES research involving adolescents. Results from the current study point to novel findings, namely that (1) parents of adolescents with TS were observed to use both supportive and unsupportive ES behaviors to socialize emotions during a conflict discussion task with their adolescents, and (2) parent ES behaviors were significantly related to adolescent psychological symptoms after adjusting for adolescent sex, in support of our hypotheses. Overall, the current study provides important implications and future directions for research that may lead to novel interventions in TS families.

Results revealed that parents of adolescents with TS were observed engaging in a range of supportive and unsupportive ES behaviors. Few studies have examined parent ES behaviors in adolescents broadly, and those that have used a wide range of methods and conceptualizations to assess ES behaviors, likely lending to inconsistency in findings (e.g., Breaux et al., 2022; Lougheed et al., 2020). In the current study, parents were observed to use both supportive and unsupportive ES behaviors during the conflict discussion task when rated by trained coders. Additionally, parents were observed to display the full range of scores for unsupportive ES behaviors and a similarly wide range for supportive ES behaviors. These findings in adolescence are encouraging evidence for the continued salience of parenting behaviors, as much less research on ES has focused on this developmental period. Altogether, the current study evidenced that parents display a broad range and varying levels of supportive and unsupportive ES behaviors when interacting with their adolescents with TS, highlighting the importance of examining parent ES behaviors during the adolescent developmental period.

Additionally, our findings highlight the importance of considering measurement for both supportive and unsupportive ES constructs. In the current study, the magnitude of the correlation between supportive and unsupportive ES codes was small, suggesting these may be distinct, though potentially overlapping, domains. This parallels research using both self-report questionnaire and observational methods of ES behavior measurement (e.g., McQuade et al., 2023). Research focusing on only one of these constructs limits insights into the relationship beween parent ES behaviors and adolescent psychological outcomes. Indeed, studies investigating varying levels of both supportive and unsupportive ES behaviors in parents have shown that the two constructs interact to predict child outcomes (e.g., Lunkenheimer et al., 2007; McKee et al., 2022). While the interaction between ES behaviors was not assessed in the current study, findings collectively support the notion that supportive and unsupportive ES are distinct constructs, each of which is associated with psychological symptoms for adolescents with TS. Future research should examine how the interaction of supportive and unsupportive ES behaviors impacts adolescent outcomes in TS.

Results also supported our hypotheses that supportive ES behaviors would be related to fewer psychological symptoms in adolescents with TS, and partially supported our hypotheses that unsupportive ES behaviors would be related to greater symptomology. Specifically, greater observed parent use of supportive ES behaviors was significantly related to fewer internalizing and externalizing symptoms in adolescents after controlling for adolescent sex, demonstrating medium effect sizes. In contrast, greater observed parent use of unsupportive ES behaviors was significantly related to greater internalizing, and marginally related to greater externalizing, symptoms in adolescents after controlling for adolescent sex, with medium effect sizes. These results in TS mirror those found in other neurodevelopmental populations, specifically youth with ADHD (e.g., Breaux et al., 2018, 2022; McQuade et al., 2021; Melnick & Hinshaw, 2000; Oddo et al., 2020). While previous studies have found mixed results for unsupportive ES behaviors (e.g., Breaux et al., 2018; McQuade et al., 2021), the current study found support for a positive association between parent unsupportive ES behaviors and adolescent psychological symptoms. Importantly, the training of ES behaviors is a modifiable target which may improve clinical outcomes in adolescents with neurodevelopmental disorders (England-Mason et al., 2023), and our findings suggest that it may be beneficial to incorporate parent training in behavioral interventions for adolescents with TS.

The current study did not find significant relationships between adolescent tic severity with either parent ES behaviors or psychological symptoms. The former suggests that parent ES behaviors may not be influenced by parent frustration or distress related to adolescent tic behaviors. While this is the first study to examine parent ES behaviors in the context of TS, the findings suggest that these behaviors may not hold unique salience in this population. Nevertheless, the significant associations between parent ES behaviors and adolescent psychological symptoms are consistent with prior literature in other populations (Eisenberg, 2020) and highlight the importance of parental sensitivity and emotional support in adolescence. Further, no significant association was found between adolescent tic severity and psychological symptoms, which contrasts previous research linking greater tic severity to increased psychiatric symptoms (Eddy, Cavanna, et al., 2011; Huisman-van Dijk et al., 2019). This discrepancy may reflect our small sample size and the somewhat limited variability in tic severity scores. In this study, the average total tic severity score was in the moderate range, and two-thirds (66%) of the sample were classified as having moderate tic severity (YGTSS scores between 11 and 27). While nearly one-quarter (24%) were classified as having marked tic severity (YGTSS scores between 28 and 43), no adolescents met criteria for severe or extreme tic severity. It is possible that the relationships between tic severity with parent ES behaviors or adolescent psychological symptoms may only be present at higher levels of tic severity. Adolescents with mild-to-moderate tic severity may have lower levels of tic-related impairment, which could include fewer behavioral problems that may influence parent ES behaviors. Alternately, adolescents with severe tic severity may experience greater impairment, and this could exert greater influence on parent ES behaviors or adolescent psychological symptoms. However, due to the small sample size and limited range of YGTSS scores, we were unable to examine these relationships across varying levels of tic severity in the current study. It will be important for future research with larger, more diverse samples to better understand how tic severity may influence parent ES behaviors and adolescent psychological symptoms in TS.

Findings from the current study should be interpreted with caution due to several limitations. First, our small sample size of 29 parent-adolescent dyads restricted statistical power and limited the ability to conduct more complex analyses. Specifically, while there is some evidence that parents may socialize emotions differently based on sex (e.g., Brown et al., 2015; Garside & Klimes-Dougan, 2002), our sample size and limited number of fathers (*n* = 5) precluded the examination of sex differences in parent ES behaviors. Further, our small sample size also precluded us from conducting moderation analyses examining the influence of tic severity on ES behaviors and interactions between supportive and unsupportive ES behaviors, as there is also evidence that supportive and unsupportive ES behaviors interact to differentially predict child outcomes (e.g., Lunkenheimer et al., 2007; McKee et al., 2022). Additionally, it is important to note some results were borderline significant, particularly the relationships between unsupportive ES behaviors and psychological symptoms in both correlation and regression analyses, and the relationship between supportive and unsupportive ES behaviors approached but did not reach statistical significance. As such, these results should be interpreted with caution. Second, our sample predominantly identified as White and non-Hispanic, which may limit generalizability of these findings. It is possible that tic severity, parent ES behaviors, or adolescent psychological symptoms may vary in other sociocultural contexts (Nielsen et al., 2017). Third, the current study did not statistically correct for multiple comparisons. This decision aimed to maximize statistical power, although we acknowledge this approach increases risk for Type I error. We did limit the number of statistical comparisons to minimize this risk. However, this in turn precluded inclusion of multi-informant assessment of adolescent psychological symptoms. Parent- or teacher-report may provide a more comprehensive understanding of adolescent psychological symptoms, and we encourage future research to prioritize multi-informant assessments. Fourth, the current study was cross-sectional in nature, which limits any inferences about causality between parent ES behaviors and adolescent psychological symptoms. Lastly, this study did not include a comparison sample, so implications about potential differences between families with and without TS cannot be drawn. An important direction for future research may be the inclusion of neurotypical adolescents or adolescents with another neurodevelopmental disorder as a comparison sample to strengthen conclusions regarding parent ES behaviors in families impacted by TS.

There are several critical implications for future interventions and research directions based on this study. Foremost, because this study showed that parent ES behaviors are associated with clinically relevant outcomes in adolescents with TS, parent training in ES behaviors may be an effective intervention to mitigate psychopathology in adolescents with TS. Further, interventions may highlight the importance of differential associations between supportive and unsupportive ES behaviors, and may focus on teaching parents to both increase supportive and decrease unsupportive ES behaviors. Future experimental and intervention research should examine how modifying parent ES behaviors (i.e., increasing supportive and/or reducing unsupportive ES behaviors) influences adolescent psychological symptoms. Additionally, it is important that future research assesses adolescent and parent characteristics related to parents’ use of ES behaviors (e.g., Breaux et al., 2016; Root & Rasmussen, 2017; Thompson et al., 2020) to understand other factors that underlie or predict the ES behaviors with which parents engage. Lastly, the influence of parenting behaviors on adolescent tic severity and comorbid psychopathology should be examined longitudinally in TS. It is possible that parental knowledge about TS may moderate the levels of supportive and unsupportive ES behaviors they use, and future research should consider examining differences in ES behaviors between families with a recent TS diagnosis and those with a remote TS diagnosis.

In conclusion, the current study revealed several important findings regarding the use of parent ES behaviors in the context of both adolescence and TS. Specifically, results revealed significant associations between parent observed supportive and unsupportive ES behaviors and adolescent self-reported internalizing and externalizing symptoms in TS. These findings are novel in the context of TS and extend that of prior work in other neurodevelopmental disorder populations. Future research may strive to investigate parenting in TS in longitudinal studies and examine the modifying effects of additional factors such as parent sex, family knowledge about TS, and adolescent tic severity. Potential implications for interventions include greater involvement of parents in treatment for TS and teaching skills about both supportive and unsupportive ES behaviors.

## Data Availability

The data surrounding the findings of this study are available upon reasonable request from the corresponding author.
